# How Can Clients Improve the Quality of Transport Infrastructure Projects? The Role of Knowledge Management and Incentives

**DOI:** 10.1155/2013/709423

**Published:** 2013-10-22

**Authors:** Abukar Warsame, Lena Borg, Hans Lind

**Affiliations:** Real Estate and Construction Management, School of Architecture and Built Environment (ABE), Royal Institute of Technology, KTH, Brinellvägen 1, 100 44 Stockholm, Sweden

## Abstract

The aim of this paper is to argue for a number of statements about what is important for a client to do in order to improve quality in new infrastructure projects, with a focus on procurement and organizational issues. The paper synthesizes theoretical and empirical results concerning organizational performance, especially the role of the client for the quality of a project. The theoretical framework used is contract theory and transaction cost theory, where assumptions about rationality and self-interest are made and where incentive problems, asymmetric information, and moral hazard are central concepts. It is argued that choice of procurement type will not be a crucial factor. There is no procurement method that guarantees a better quality than another. We argue that given the right conditions all procurement methods can give good results, and given the wrong conditions, all of them can lead to low quality. What is crucial is how the client organization manages knowledge and the incentives for the members of the organization. This can be summarized as “organizational culture.” One way to improve knowledge and create incentives is to use independent second opinions in a systematic way.

## 1. Introduction

Cost, time, and quality are the three main dimensions when project results are evaluated. In this paper, the focus is only on the last of these three: how can quality be improved and what can be done to avoid quality problems? As discussed in Warsame [1] quality in relation to construction projects can be given different meanings. A first distinction is between quality of product and quality of process. In this paper, the focus is on the quality of the product. Another important distinction is between quality as an absolute concept in relation to certain standards and quality as a relative concept where quality is related to what the client had ordered and/or what the client reasonably could expect, given the price they are willing to pay. In this paper quality is used in this relative sense, which more generally can be described as how to get “value for money.”

The debate about quality improvement is going on at different levels: from the more practical to the more general. Choice of procurement method is one question on the more practical level [2], while knowledge management and creation of incentives in the organization are questions of the more general level [3]. A main notion in this policy paper is that the more general level is the most important. 

The focus of this paper is on infrastructure projects where there is a large public client like the Swedish Traffic Administration which is responsible for both roads and railways and has an operating budget of 50 billion SEK for 2010 (around 5 billion €). 

We will argue for three statements: the first one is that “quality is up to the client,” and this is developed further in Section 3 below. The second statement is a negative one and says that there is no clear relation between *procurement type* and quality (see Section 4). The third and more positive statement concerns two crucial interdependent dimensions for getting high quality: *knowledge* and *incentives *(see Section 5). The famous Swedish builder Olle Engkvist wrote the following in a book from 1949 (translated by authors):

*That a low-quality building ever is constructed depends on that the builder either lacks one or several of the necessary qualifications for the trade, or that the profit motive is so dominating that it overshadows all other interests*. [5, page 9]**

In a governmental organization, it does not have to be the profit motive that creates problems, and the term can be exchanged for “ulterior motives” in general, for example, political ones. This is developed further in Section 5. Concluding comments can be found in Section 6.

## 2. Method and Conceptual Framework

The paper tries to synthesize both theoretical and empirical results. There is very large literature in this area, but we hope that the selection made covers the most important arguments and results. Our aim is to try to present theoretical and empirical arguments that make the statements presented above convincing. Future debates will determine to what extent we have succeeded. From a broader methodological perspective the approach is closest to the ideas of Karl Popper as the propositions presented can be seen as “conjectures” that, according to our view, have so far not been refuted [6].

The theoretical framework used is general contract theory and transaction cost theory, where assumptions about rationality and self-interest are made and where incentive problems, asymmetric information, principal agent problems, and moral hazard are central concepts. The concepts and ideas from these theories will be presented a little more in detail in Sections 4 and 5 below when procurement is discussed.

## 3. Why Quality Is up to the Client?

It is possible for a nonexpert to know the quality of a new car reasonably well, but it will be argued here that a traffic authority cannot rely on “the market” if they want to build a road with a certain quality. 

The car is typically produced in a large volume in a plant with strict control of the production process. The company has produced cars over a number of years. As the life of a car is rather short, it is possible to collect information quickly about the quality of a certain brand and a certain model. In a country like Sweden where cars have to be inspected every year, a lot of third party data are published on faults in all car models. The result of this is that the household does not have to be an expert or even consult an expert when they buy a new car (with old used car the situation is different, e.g., [7]). Tests of new cars are also regularly published in both general and specialized newspapers.

A section of, for example, a road is instead typically

- produced “in the field” where surveillance can differ considerably and where external factors can affect the quality of a specific construction;
- produced by a group of people that change more or less from project to project. If company A does a good job in project 1 in region 1, it does not mean that company A will do a good job in project 2 in region 2 since different group of persons will produce the road. Big construction companies are typically rather decentralized (see e.g., [8]);
- a product where it takes a rather long time to find out if there are quality problems, and it might not be the case that company A today is as bad as it was maybe 10 years ago when the road was constructed.

All this means that the mechanism used by the buyer of a car is difficult to use for a client responsible for an infrastructural project. In the rest of the paper, it is therefore assumed that the market feedback mechanisms in infrastructure construction projects are too weak to be relied upon only. The client must then use more direct methods to assure that a certain quality will be delivered. 

This view of the role of public client has been underlined by several authors, even if the theoretical background to their statements is not so clear. The procurement of these assets, and proper operation and maintenance require a client workforce with strong competence, skills, and experience. Ward et al. [9] stress that client's stock of experience and advice received are crucial. Public clients need to maintain enough skilled and competent workers and management in order to manage risks and safeguard public interest of construction projects [10, 11].

## 4. Procurement Types Have No Determinate Consequences

### 4.1. Design Responsibility and Quality

The tendency in many countries seems to be moving away from making the design in-house to using external consultants. There can be several explanations for this; for example, fluctuations in the number of projects make it difficult to employ an in-house workforce, and this problem is increased when the workforce becomes more specialized. It might also be more difficult to create strong incentives in an in-house organization. In Warsame [12], there is a more general discussion of the trend away from both in-house technical specialists and in-house construction workforce among developers and public authorities.

Independent of the reason for this development, the discussion here will focus on a comparison between the case where the *client* hires a technical consultant to doing the detailed design and the case where the *contractor* works together with a technical consultant and do the detailed design. Notice that the arguments against the client/developer having their own staff also are relevant for the question whether the *contractor* has an in-house staff or not. This means that it might be the same companies and individuals that make the detailed design independent of whether the client or the contractor is responsible for the design. The question of the skills of the technical consultants doing the work should then not be an argument that points in a specific direction when it comes to who should be responsible for the detailed design.

A classical work on economic organization, Milgrom and Roberts [13], describes the general problems in an economy in terms of achieving *coordination* and creating *incentives*. These aspects seem highly relevant for the choice of who should do the design.

- From a *coordination* perspective, the rational choice would be to let the contractor be responsible for the detailed design as the design then can be adjusted to the technical competence of the contractor and the design can be carried out with more knowledge about the construction process.
- From an *incentive* perspective, the rational choice would be to let the client be responsible for the design. If the technical consultant works for the contractor, there should be pressure on the consultant to choose cheaper solutions within the limits set by the standards laid down by the client. It might be difficult to know and observe the exact quality of all technical alternatives, and some incompleteness or vagueness can be expected in the client's standards, and this opens the door for the contractor to influence the design in the direction of cheaper solutions with somewhat lower quality.

A counterargument against this is that stronger incentives for the contractor to choose the “right” solution might be created if the contractor also is responsible for operation and maintenance. This will be discussed more in detail below, and for now it is, assumed that the contract only concerns the construction phase.

The implication of the arguments above is, of course, that things might go wrong in both alternatives. If the client is responsible for the detailed design and does not have enough knowledge of the production phase, there will arise a need for redesign and costly adjustments. CIOB [14] underlines the importance of completeness and clarity of client's needs and objectives when they are responsible for the design phase. The overall quality might also suffer if the design is not adjusted to the skills of the contractor. On the other hand, if the contractor is responsible for the design, there might be a risk that alternatives with lower cost and quality are chosen if the specifications and the monitoring by the client are imperfect.

A client who is aware of these potential problems can however mitigate them, at least partly. If the client is responsible for the detailed design they—and/or the technical consultant—may build up knowledge of the construction phase in order to reduce the risk for coordination failures. If the detailed design is made by the contractor, the client may be more careful with the specifications, or for some components where quality is difficult to evaluate expost, the client might simply say that this is the component that should be used. If the reputation of the contractor is important for the choice of contractor in forthcoming projects, it might also be risky for the contractor to choose a cheaper alternative with lower quality as this might reduce the probability of future work for the client.

We also see here that the line between the alternative procurement types becomes vaguer. A knowledgeable client may, even if they are responsible for the detailed design, leave some room for adjustments of the design after the contractor is chosen in order to take advantage of the comparative skills of the chosen contractor. On the other hand, if the client's specifications become more and more detailed in the case where the contractor is responsible, then the room for the contractor in the design stage might be rather small, even if they formally are responsible for the design.

### 4.2. Quality and the Integrating of Construction and Operation/Maintenance

In recent years, a number of theoretical studies have pointed out that bundling construction and operation/maintenance can lead to higher efficiency, as is done in, for example, different forms of public private partnering projects (PPP). No distinction will here be made between different forms of contracts where construction and operation/maintenance are bundled, for example, differences in how the project is financed and how the contractor is paid.

Bennett and Iossa [15] and Martimort and Pouyet [16] pointed out that this type of bundling leads to higher efficiency because coordination between construction and maintenance can be improved. The design can in a better way take into account consequences during the operation/maintenance stage, and this reduces life-cycle cost. Better knowledge of how the construction works have been carried out can also lead to operation/maintenance measures that are better adjusted to how the facility was built.

Another important feature of these long-term bundled contracts is that they, at least partly, are formulated in performance terms. The client sets up a number of performance criteria that the facility should fulfill over time, and the payment to the contractor is dependent on that these conditions are fulfilled.

The potential from a quality perspective of contracts that bundle construction and maintenance is clear: the responsibility for supplying the quality that is stipulated in the contact is completely in the hands of the contractor, and their payment is dependent on that they produce a service with this quality.

As argued in, for example, Lind and Borg [17], there are a number of general problems with realizing this potential in bundled contracts, for example, how contractors can collect and transfer knowledge within their organization about how operation and maintenance costs are related to how the facility was constructed. Here the focus will, however, be on issues more directly related to the quality of the object.

The *first* main problem is the possibility of describing the quality that the client wants in a way that is possible to measure in a rather objective way. Robinson and Scott [18] point out that the description of services in PFI/PPP projects typically lists a large number of characteristics and that this has still not been enough to get the contractor to produce what the client really wanted. The quality of the facility, in some dimensions, was not the expected one because it was difficult to write a contract that was complete enough. Their general message is that describing service quality is very difficult and that a lot of resources must be put into specifying service quality. Guo et al. [19] also points out measurement problems in a contract with functional demands. 

A *second* contractual aspect that can be problematic in performance based contracts is the verifiability of the specified characteristics. Lind and Mattsson [20], evaluating an experiment with performance based bridge maintenance, show that there were often disagreements between client and contractor about whether the characteristics specified in the contract were fulfilled or not.

In general, one can say that writing complete long-term contracts is a very challenging task and that there are bound to be mistakes or lapses that can lead to lower quality than expected in the objects; see, for example, Milgrom and Roberts [13] for a discussion on conditions for complete contracts and why they are difficult to fulfill. 

A *third* general problem with long-term bundled contracts is what happens over time. The theoretical studies typically assume that there is a completely binding contract and that the contractor has a real long-term responsibility for the object. There are several problematic assumptions behind statements like these.

The first assumption is that there will be no renegotiations of the contract. Renegotiations have been common in Latin American PPP projects [21]. Even if they focus on payments and cost-overruns, the same problem might occur concerning certain quality aspects. A contractor with good political connections may be able to renegotiate and get the client to accept a lower quality than the one originally stipulated.

A second assumption is that the contractor will not sell the project. In recent years, a number of infrastructure funds have been started that are buying PPP projects (see, e.g., [22]). Initially the project is owned by a construction company, but when the project is completed, it is sold to an investor. This might seem logical from a comparative advantage perspective as the construction company has their advantages in the initial stages of the project. If the contractor plans to sell the project, the incentives for the contractor to choose techniques that minimize life-cycle costs are reduced as there will be asymmetric information between the contractor and the new investor. The contractor might build with lower quality in dimensions that are difficult to evaluate for a buyer, and this creates higher operating and maintenance costs later on. Entering PPP projects is also risky, and the private party might have underestimated the costs and/or overestimated the incomes. Studies have shown that when an actor gets under financial pressure they tend to reduce quality (see [23] for an example from the retail sector).

### 4.3. Concluding Discussion of Procurement Types

The proposition in this part of the paper—that quality is independent of choice of procurement type—has, as far as we know, not been formulated as clear and straightforward as here, but there are statements that are very much in line with our views. Many authors discuss in what situations a certain procurement type is best, but typically the statements are vague and very guarded, which is what we should expect given the views formulated above. 

Ashworth [24] writes

*Individual experiences, prejudices, vested interests, familiarity, the need and desire for improvement are all factors that have helped reshape procurement in the construction industry. (page 298)*

*The arguments for engaging either a consultant or a constructor as the client's main advisor or representative are to a large extent linked with tradition, fashion, loyalty and the satisfaction or disappointment with a previous project. (page 295)*

This means that one client might go from procurement type A to procurement type B in order to increase quality, while another for the same reason moves in the opposite direction. And this should not be surprising if the direct relation between procurement type and quality is weak.

In a similar way, Lædre et al. [25], for example, writes “A client's choice of procurement method, among other factors, could be influenced by the client's familiarity and prior experience with that method as well as the level of client involvement required by the selected method.” The same point is made in HM Treasury [26]. Molenaar and Songer [27] underline the role of public agency's staff and experience to the success of projects procured in DB delivery method, which implies that during some circumstances this method might work well but not in other circumstances.

In the literature, one can find statements that each procurement method has advantages and disadvantages and that they are suitable for different situations. Accordingly, the proposition can be formulated in other ways. The most general one is to say that there is no quick fix when it comes to improving quality in infrastructural projects. In other words, there are no simple deterministic relations between underlying factors and the quality that will result in a project. Kwame et al. [2] found that rework causes do not differ relative to various procurement methods. Thus, there is no procurement method that guarantees a better quality than another. 

It has been argued that if a client has low technical competence, then choosing design build (DB) procurement would be better as the client then only has to specify the characteristics of the final product. The first counterargument is that if you do not have technical competence, it will be very difficult to specify all relevant characteristics of the object. The second counterargument would be that the client could just as well contact a technical consultant and make the detail design together with them, and then use a DBB procurement. A client that has good relation with a technical consultant would probably choose the second alternative while a client with good experience from working with a specific contractor would choose the first option. 

## 5. The Importance of Knowledge Management and Incentives

In the last section, it was argued that choice of procurement type will not determine quality. In the following sections, the focus is on what we think is important: knowledge management and creating the right incentives. 

### 5.1. General Definition and General Types of Knowledge

According to Nonaka and Takeuchi [4], knowledge can be classified as explicit and tacit. Explicit knowledge is described as knowledge that can be precisely and formally articulated. It is easily codified in different formats that would allow for documentation, transfer, sharing, and communication. Tacit knowledge is a knowledge that comprises experience, and work knowledge that resides only with the individual and is difficult to formally articulate. Pathirage et al. [28] claim that tacit knowledge based on skills, experience and talent of people is considered to be relatively unexplored and underutilized when compared to the work on explicit knowledge. Information technology tools often address the explicit knowledge while non-IT tools address the tacit knowledge.

This distinction between knowledge of different types has shaped the strategies of *knowledge management* followed by different organizations [29]. There are numerous definitions of knowledge management, but here the definition of Scarbrough et al. [30] cited in Al-Ghassani et al. [31] will be used. It combines both the process and outcome perspectives of knowledge management. It states that *knowledge management is any process or practice of creating, acquiring, capturing, sharing, and using knowledge, wherever it resides, to enhance learning and performance in organizations*. 

**Figure 1:**
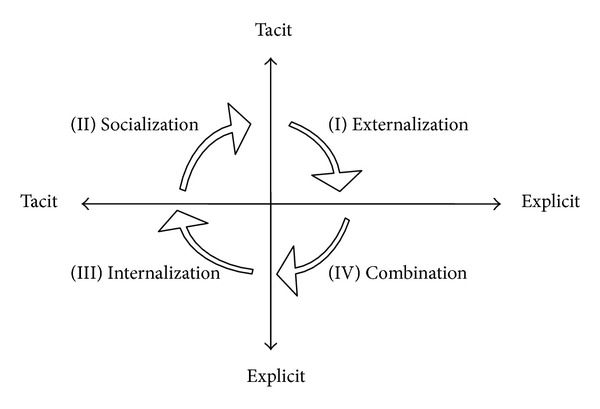
Knowledge conversion modes [4].

C. Gore and E. Gore [32] suggest a strategy of organization's knowledge management that combines the use of current explicit knowledge, capturing new explicit knowledge, and externalization of tacit knowledge. Egbu and Robinson [33], based on Nonaka and Takeuchi's theory of knowledge creation, also describe four distinct modes of interaction between tacit and explicit knowledge: socialization, externalization, internalization, and combination (see Figure 1). 

A designer's explanation of design concepts to client is tacit to tacit interaction, and it takes place through the process of *socialization* (2nd quadrant). Apprenticeship and mentoring schemes between senior engineers mentoring junior engineer is another example of tacit to tacit interaction. Such experiential knowledge is nurtured through shared experience and continuous interaction [33]. Next the designer uses manuals on design standards and interprets these explicit documents to a unique design that could satisfy the needs and the requirements of clients. This knowledge transformation from explicit to tacit is termed *internalization* (3rd quadrant). When the architect/designer translates a design concept into sketches in order to explain to the client, the architect transforms tacit knowledge to explicit and is called *externalization* (1st quadrant). Another example of externalization process is when a junior engineer transforms the tacit knowledge that he or she gained from senior engineer through the socialization interaction to explicit knowledge. The 4th quadrant represents the *combination* process where explicit to explicit interaction takes place. Knowledge is created through integrating and processing of different documents such as design briefing and sketches, performance and standard specifications, and estimates and contract requirements. 

Both socialization and externalization are required to create an ever-growing body of organizational routines [34]. Quality circles and task forces that are widely used to enhance total quality and continuous improvement are examples of externalization processes creating firm specific routines [34]. 

### 5.2. Knowledge Management in Practice

From a knowledge management perspective, the following components can be identified and are, as we see it, all necessary conditions for an authority to be able to reach high quality.

1. *The building up of long-term explicit knowledge through research. *The authority needs research in order to improve their knowledge. It has to be active in procuring and/or doing their research on issues of long-term importance. The Swedish Traffic Board, for example, procures result from a large number of researchers every year.
2. *The building up of knowledge through tests*. in order to become more sure about how a certain system would work, various solutions have to be tested in practice, and systematically documented.
3. The *building up of knowledge through cooperation with foreign experts and consultants. *Scanningwhat others do and pooling knowledge with other organizations and firms are examples of this. There is for example, long-term cooperation between Nordic traffic authorities and broader cooperation within various EU-projects.
4. *The building up of a systematic management of the organization's own experience.* As the typical transport authority handles a large number of procurements and projects, it is important to underline the need for continuous monitoring of how different projects worked out.

### 5.3. The Importance of Incentives

Many organizations have recognized that the success of knowledge management depends on people and their behavior [35]. Employees must be sufficiently motivated to share knowledge [36]. Teerajetgul and Charoenngram [37] emphasize how incentives or reward could significantly affect internalization of the knowledge creation process. They state that the vision and aspiration of construction managers in applying creativity in on-site knowledge practices play crucial role on the strength of knowledge management. The question is how to create incentives for individuals to build up, share, and reveal relevant knowledge and information that could be useful in improving the performance of projects. Osterloh and Frey [34] suggest that organizational forms that emphasize participation and personal relationship are needed. However, the answer for the above question also requires a deeper discussion about incentives, and here the economic approach is the starting point.

Milgrom and Roberts [13] attempted to synthesize management theory and economic theory, but the base is clearly in economic theory (microeconomic theory, contract theory, and transaction cost theory). One central starting point in Milgrom and Roberts' economic approach is that the basic unit for understanding how organizations work is the individual. One has to understand the incentives of individuals in different parts of the organization in order to understand how the organization works. 

Incentives can be of many different kinds. There might be internal incentives where people do certain things just because they want to do a good job and sustain a certain image of themselves. Ellingsen and Johannesson [38] discuss this from this perspective of why people, for example, give tips to taxi-drivers when travelling in a foreign country, where they never will meet that driver again. Incentives can also concern career opportunities: if I do certain things today, it increases the probability to come to a higher position and get more money, a more interesting job, and/or more power in the future. Of course, incentives can also concern short-run gains including both economic bonuses and positive feedback from colleagues and superiors.

#### 5.3.1. Precondition  1: Knowing Who Did What

An important precondition for creating stronger incentives is that it is possible to know who did what. Carrillo [39] argues that peer recognition of employee's contribution and acknowledgement of individual's achievement such as manager of the year award has a more sustainable impact than financial reward. It is in this respect interesting to compare, for example, what information is presented when a movie is ready and what information is presented when a construction project is ready. At the end of the movie, hundreds of names are presented giving information about who did everything from directing, producing, and acting, to being the driver and the assistant to the actors. This information is available “forever.”

But assume that after a few years someone wants to know who did what in a construction project? Finding out who was the architect and what companies were responsible for what might not be so difficult, but that does not apply, for example, for the site manager and also for who was responsible for installing the electric system and who painted the walls inside the building? An important precondition for creating stronger incentives during all the different stages from planning to construction would then be a systematic recording of who did what in a project. 

#### 5.3.2. Precondition 2: Repeated Games

One of the strongest ways to create incentives is through “repeated games” (see e.g., [40]). If you do a good job, the probability of getting hired again and getting a better and better paid assignment increases, and the opposite happens if you do a bad job. In a number of sectors in the economy, this type of incentive mechanism is the dominating one. The movie industry is an obvious example mentioned above. Lindahl and Leiringer [41] analyze project management in the event industry where teams are put together for each specific event. One central criterion is that if a person did a good job at an earlier event, then that person is trusted to be responsible for setting up the event. 

Looking at infrastructure construction from the perspective of repeated games, it is possible to see several problems. The first concerns the relation between the client and the contractor in the public sector. In a comparison between procurement by private and public clients in the housing sector, one result was that the private clients preselected 2-3 companies according to their earlier experience and knowledge of the companies (see, e.g., [42]). If the client was dissatisfied with a company, then this company was deleted from the list. The public client worked under the Law of Public Procurement and had open tenders, and they had to follow strict criteria both in the prequalification stage and when choosing contractor. 

The second problem from the perspective of repeated games is that on the client side the staff on all levels is hired by standard employment contracts with fixed wage. The level of employment protection is high in countries like Sweden which means that the direct difference for the employee between making a good job or not from the perspective of long-term quality can be small. The question is then how incentives can be strengthened *within* a public authority.

#### 5.3.3. Incentives in a Public Administration

The purpose of this section is primarily to give examples of how things should not work and how they might work. The following is a stylized example of a structure where incentives for quality are not so strong (the example is partly inspired by the discussion in [43, Appendix  1].

*Election time is closing in and it is very important for the government to show both that they are starting up projects and finalizing projects that are important for winning the elections. Projects then have to start up quickly without enough preparations. The civil servants working with the cases, somewhat disillusioned from earlier cases, also knows that there will be a number of changes and adjustments later in the project, so there is no point in putting in maximum effort concerning the design in an early stage. Civil servants that might have protested against certain “bad” decisions earlier are seen as troublesome and causing delays and have more problems to get promotions. Most employees remain quiet and shrug their shoulders knowing that problems will come later.*

The importance of things working smoothly and of avoiding conflicts can also affect the work during the construction stage, for example, saying yes to proposals from the contractor even if there is a risk of lower quality [44]. Warsame [1] describes different “decision styles,” and several of these underline the importance of consensus and avoiding conflicts, and this can lead to client representatives accepting lower quality than actually contracted and/or higher risks for quality problems. 

All these things can be described as part of a “company culture,” and *creating the right culture is important for the long-term quality of the projects*. The culture in the authority, together with their competence, will affect the incentives for consultants, contractors, and contractors, that—to simplify somewhat—do what it is necessary to survive in the market rather than what is essential to the long-term quality of the project.

In a public authority, the politically chosen board, and leading politicians on all levels, is of course in the end responsible for how the authorities work. These “final” decision makers send out signals about what they approve of and do not approve of, and these signals behavior will affect the company culture in the authority. But, of course, also public employees on all levels have a responsibility towards the taxpayer and citizens to contribute to an efficient use of resources in the public sector.

### 5.4. “Second Opinions” as a Crucial Instrument

One way to both improve knowledge and creative incentives is to use “second opinions” from external actors in a systematic way during all stages of an infrastructural project. This is already done in some countries to avoid cost-overruns in large infrastructure projects (see [45, 46]), but broadening this to include other aspects including possible quality problems seems to be one promising way to both improve knowledge on possible consequences of various changes and create incentives. Knowing that your proposal will be evaluated by an external reviewer and that this will be documented and be available for others should increase effort and make it easier to evaluate the quality of both departments and individuals. 

This will increase cost and could cause some delay in the processes, but the assumption here is that the gains will be bigger than the costs. Choosing the external reviewers is also a problematic issue, and it is rather obvious that if the top management does not take the external review seriously, it will always be possible to find “yes-men.”

## 6. Conclusions

Public client organizations typically act as the owner and the party who instigated a project for the benefit of society. In order to ensure that a desired project performance is achieved and provide better infrastructure projects, it has been argued that the public client's “organizational culture” is the most important, and the core of this culture should be a focus on knowledge management and creating incentives, for the desired behavior in the organization. A central instrument for top management to signal this is to systematically work with “second opinions” from independent actors in a form adjusted to the size and specific stage of the process. 

This also implies that, for example, the choice of procurement types cannot by itself improve project performance. Without knowledge and the right incentives it is unlikely that any procurement type will lead to high quality results and an organization with the right knowledge and incentives can adjust any procurement type to the situation and make it work.

Finally, let us repeat that these statements are not “proven facts” but conjectures that we have argued are consistent with both theoretical and empirical studies concerning quality in infrastructure projects.
